# Barriers and facilitators to telerehabilitation implementation: a mixed-methods study of German physiotherapists

**DOI:** 10.1186/s12913-026-14636-6

**Published:** 2026-05-09

**Authors:** Marco Stahn, Martin Roemhild, Christian Kopkow, Anne-Kathrin Rausch

**Affiliations:** 1 Zurich University of Applied Sciences (ZHAW), Institute of Applied Sciences Physiotherapy, Winterthur, Switzerland; 2 Brandenburg University of Technology Cottbus – Senftenberg, Faculty 4 - Institute of Health, Senftenberg, Germany; 3 Institute of Evidence-Oriented-Medicine – FIEOM.de, Emmerthal, Germany

**Keywords:** Barriers, Facilitators, Mixed-methods, Telehealth, Teletherapy, Telerehabilitation, Physiotherapy

## Abstract

**Background:**

The COVID-19 pandemic accelerated telehealth service adoption in physiotherapy, including telerehabilitation (TR). However, the extent of TR use and factors influencing its implementation in Germany remain unclear. This study aimed to evaluate TR use in physiotherapy (during COVID-19 lockdowns, current use, future intentions, conditions treated, content, setting, and session type) and identify barriers and facilitators from physiotherapists’ (PTs) perspectives.

**Methods:**

A mixed-methods sequential explanatory design was employed, combining a cross-sectional online survey (*n* = 152) with two focus group interviews (*n* = 9). The survey collected data on demographics, TR use, barriers, and facilitators. Focus groups explored themes emerging from the survey in greater depth. Data were analyzed using descriptive statistics and content analysis, integrating quantitative and qualitative findings. Quantitative and qualitative findings were integrated through explanatory linking and joint display in a comparative analysis table.

**Results:**

TR use peaked during COVID-19 lockdown (32.26%) but decreased to 18.06% by October 2022, with 26.45% intending future use and 43.87% considering it. Among TR users, musculoskeletal conditions were most commonly treated (75%), followed by sports (38%), pulmonology (33%), and neurology (27%). The primary barrier was lack of physical examination (74% agreement). While technical challenges were not reported as a major barrier in the survey, interviews revealed significant concerns about insufficient internet bandwidth and technical infrastructure. Common reasons for using TR included promoting patient self-management (78% agreement) and broadening therapy options (69% agreement). Qualitative data identified additional implementation facilitators, including structured implementation processes, appropriate technical infrastructure, and patient involvement in decision-making.

**Conclusion:**

While TR implementation in German physiotherapy shows growth potential, several barriers currently limit its adoption. Successful implementation requires addressing PTs capabilities, knowledge gaps, professional identity concerns, and environmental factors. Addressing these issues could enhance patient care quality, increase service accessibility, and advance healthcare delivery models.

**Supplementary Information:**

The online version contains supplementary material available at 10.1186/s12913-026-14636-6.

## Background

The global healthcare landscape has witnessed a significant surge in telehealth services, particularly in the wake of the COVID-19 pandemic [1]. However, this rapid implementation has been accompanied by the emergence of various challenges to telehealth use [2]. In Germany, as in many countries worldwide, physiotherapists (PTs) working in outpatient settings have primarily encountered telehealth in the form of telerehabilitation (TR) [3].

During the pandemic, TR was first used as a temporary solution. Now, it’s officially recognized in German healthcare regulations and can be covered by health insurance [4]. The directive defines TR as “synchronous communication between a healthcare provider and a patient, primarily by means of online treatment via video transmission in real time” [5, 6]. TR allows patients and PTs to interact through video calls in real-time and has been described in previous literature [6, 7].

While TR has been officially recognized and integrated into the German healthcare system, allowing patients to access remote therapeutic services beyond the pandemic, this regulatory inclusion was not accompanied by scientific evaluation [8]. Consequently, there is a lack of understanding regarding the extent and manner of TR use by PTs in clinical care.

Recent studies have demonstrated that TR is equally effective as in-person therapy for many common musculoskeletal conditions [9–12]. Moreover, a systematic review on the cost-effectiveness of TR suggests it could be a valuable treatment option when face-to-face rehabilitation is challenging due to logistical, resource, or pandemic-related constraints [13].

While patient satisfaction and experiences with telehealth services are consistently positive [14], PTs perspectives are more varied [15]. Although high levels of acceptance has been shown [16], many PTs express skepticism about the long-term use of TR services [16–18]. A common perception is that TR is less effective than traditional methods, primarily due to the lack of hands-on techniques, which is believed to result in poorer diagnoses and therapies [19].

Studies from different countries have identified several challenges that prevent PTs from using TR [2]. Changes in clinical practice, low confidence in technology use, concerns about depersonalization in healthcare, and issues related to patient safety and data security are primary barriers regarding the use of TR in clinical care [3, 20]. Conversely, facilitators could include sufficient education and training, appropriate technical infrastructure, and adequate remuneration [16, 17]. The uptake and use of TR may vary considerably across different countries due to different healthcare systems, national regulations, and the historical evolution of physiotherapy education.

Limited information is available about TR use in German physiotherapy. Studies indicate that German PTs are less likely to adopt TR compared to other healthcare professionals [21]. The main reasons cited include lack of technical infrastructure, insufficient training and knowledge, bureaucratic hurdles, and uncertainties regarding billing and funding [21]. The survey was conducted during the COVID-19 pandemic and included PTs, occupational therapists, and speech therapists. The timing may have influenced perceptions and practices of TR. The absence of information regarding respondents’ work settings (inpatient or outpatient) and the relatively small sample size raise concerns about the generalizability of the findings. Furthermore, while the study identifies preliminary barriers such as inadequate technical infrastructure and bureaucratic challenges, it lacks an in-depth analysis of the underlying causes and potential remedies. More comprehensive research is needed to understand TR implementation in physiotherapy practice and to develop strategies for overcoming identified barriers.

Despite the growing body of research on TR efficacy, the perspective of PTs on its use has not been sufficiently investigated in Germany [22]. Understanding these perspectives, particularly in the German context, is crucial for successful implementation and use of TR in physiotherapeutic patient management.

This study was designed to evaluate TR use in German outpatient physiotherapy and to identify barriers and facilitators from PTs perspectives.

## Methods

**Fig. 1:**
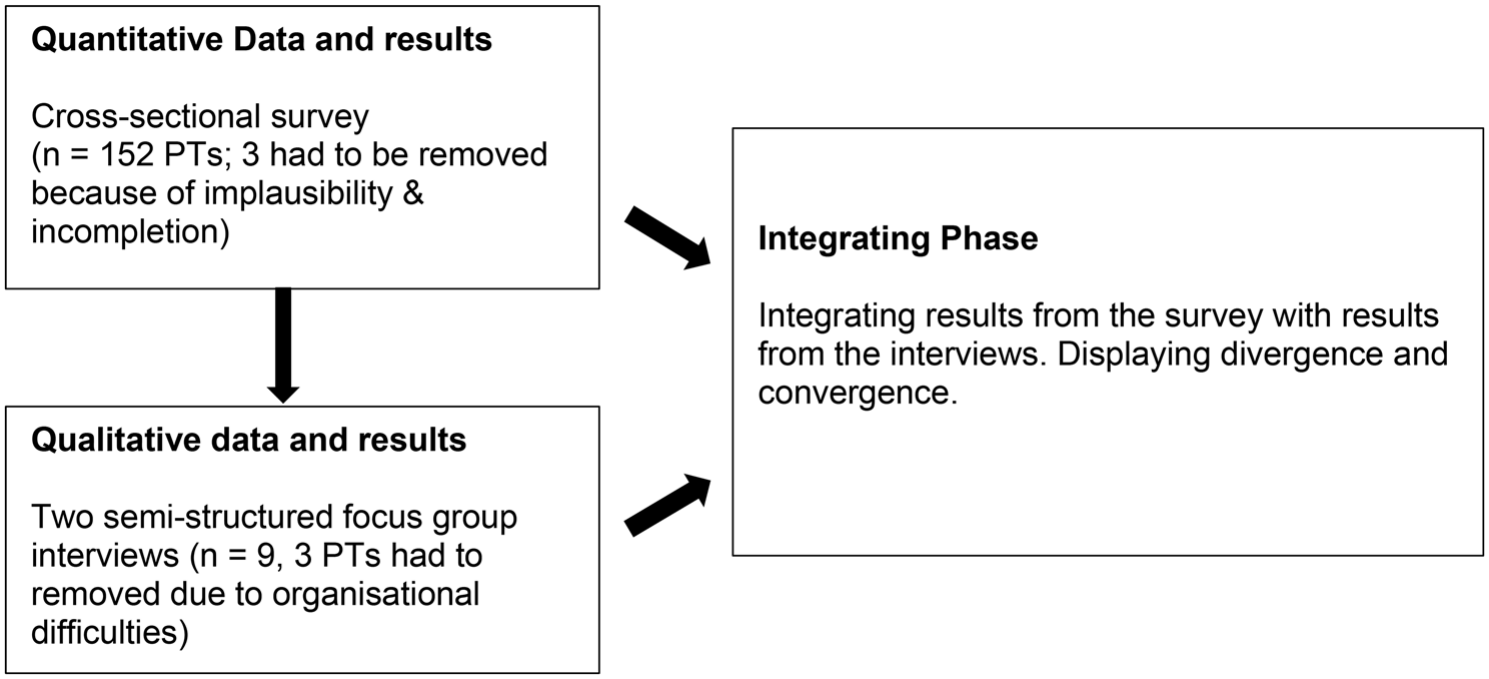
Flow of explanatory mixed-methods design

A sequential explanatory mixed-methods design study was conducted [23]. (Fig. 1) The checklist for Mixed-Methods Reporting in Rehabilitation and Health Sciences (MMR-RRS) was used for the reporting of this study [24] (supplementary material 1).

### Study design

A mixed-methods approach was chosen to address the research questions comprehensively. The quantitative strand, consisting of a cross-sectional online survey, aimed to capture broader trends in TR use among German PTs. The qualitative strand, comprising two focus group interviews, was designed to explore individual PTs perspectives and experiences with TR in depth. This combination of methods was intended to provide complementary data on both the extent and nature of TR use in physiotherapeutic patient management.

This evolution in our methodological approach reflects the iterative nature of mixed-methods research, where the interplay between different types of data can lead to unexpected but valuable insights. The equal representation of quantitative and qualitative findings in our results allows us to present a more holistic view of the current state of TR use in German physiotherapy practice, highlighting both general trends and individual experiences.

### Participants

#### Online survey

PTs (≥ 18 years) working in outpatient care in Germany were invited to participate. Recruitment was done through physiotherapy networks, newsletters, social media, and personal contacts across outpatient physiotherapy settings in Germany, including practices that provide services predominantly (minimum 50%) through statutory health insurance providers, using a “snowball” sampling technique [25]. PTs from all clinical specialties (such as musculoskeletal, neurology, pediatrics) were eligible to participate.

The aim was to recruit as many participants as possible, with a target of 500 based on comparable studies. No a priori sample size was defined given the novelty of TR research in German physiotherapy.

#### Focus group

Focus group participants were recruited from survey respondents who expressed interest. The recruitment strategy aimed for a diverse (age, gender, professional experience, attitude, specialized discipline, federal state) cohort of 9–12 PTs across Germany. Two focus groups were determined to be sufficient as similar themes emerged across both sessions. While additional groups might have provided more perspectives, scheduling constraints and data convergence supported this decision.

### Data collection & data analysis

#### Online Survey

A questionnaire was developed based on published studies on TR during the Covid-19 pandemic [21, 26]. The questionnaire consisted of five main sections:

1. Demographic Information
2. Attitude towards technology
3. Experience and Use of TR services
4. Reasons that may hinder the use of TR
5. Reasons that may facilitate the use of TR

Data collection involved single answers and multiple answers. Participants were also asked to evaluate Barriers and Facilitators on a 5-point Likert scale. Data collection occurred between August and October 2022 using REDCap [27]. The survey was accessible via a public link, without duplicate protection to enable multiple participation from shared devices. Participation was voluntary and anonymous. To ensure validity, the questionnaire used in the survey was pilot tested with five PTs representative for the intended survey population. Minor amendments to punctuation, spelling, and grammar were made based on pilot feedback. Data were analyzed using Excel Version 16.86 and R version 4.2.3.

As part of the data analyses, identified barriers and facilitators were categorized using the Theoretical Domains Framework (TDF) [28]. The TDF is a widely-used tool that helps understand factors influencing healthcare professionals’ behavior. It organizes these factors into 14 domains, such as knowledge, skills, and environmental context, making it easier to identify what helps or hinders the adoption of new practices. This framework provides a systematic method for identifying influences on healthcare professional behavior related to implementation of evidence-based practices. The categorization process involved a collaborative discussion among the research team, where each identified barrier and facilitator was mapped to the most appropriate TDF domain. This approach was chosen to enhance comparability with existing literature and provide a structured theoretical lens for interpreting our findings.

#### Focus group interviews

Focus group interviews were conducted in November and December 2022 via video conferences by the primary investigator (MS). Interviews lasted 60–90 min and were recorded with participant consent.

The interview guide was based on four main categories derived from the survey: (1) Barriers to TR, (2) Reasons for using TR, (3) Benefits of TR, and (4) Facilitators for TR. The full interview guide is available in the supplementary material 2.

Interviews were transcribed and analyzed using MAXQDA (Analytics Pro 2022) following Kuckartz’s content analysis approach [29]. Coding involved both deductive (based on interview structure) and inductive (emerging from responses) approaches. Data analysis was primarily performed by MS, with reliability ensured through independent parallel coding of 20% of the data by two researchers (MS and AKR). While a higher percentage of dual coding would have been preferable given the sample size, consistency in coding approaches was maintained through regular team discussions.

### Integration of quantitative and qualitative data

Integration of data occurred at multiple levels [30, 31]. Survey data provided the primary framework for understanding adoption patterns, while qualitative data was used to explain findings and identify additional implementation considerations. The survey identified two key elements: barriers to TR and reasons why PTs use it. While facilitating factors were not directly surveyed, they could be inferred from the reasons given for TR use. In the subsequent interviews, participants were asked to explicitly identify facilitating factors and implementation strategies. This approach allowed survey findings about barriers to be validated, while the interviews provided detailed insights into factors that support successful implementation. Survey results were discussed in focus groups to allow deeper exploration. A joint display of integrated data along with a comparative analysis is presented in Table 1.

**Table 1:** A joint display of integrated data from the survey and the focus group interviews

Topic	Survey (S)	Interviews (I)	Convergence/Divergence (C/D)
Physical Examination	Primary barrier to TR	Possible without contact; TR can replace some in-person sessions	D: S highlighted major barrier; I suggested potential to overcome
PTs as a barrier	Not prominent	Strong barrier due to reluctance/skepticism	D: I revealed insight not captured in S
Technical Challenges	Not major	Insufficient internet bandwidth is significant	D: I showed more problems than S suggested
Data Protection & Liability	Not major	Ambiguities in legal framework	D: I uncovered concerns absent in S
Remuneration	Mixed impact on TR use	Mixed impact on TR use	C: Consistent findings; complex relationship with TR adoption
Patient-Related Barriers from aPTs perspective	2nd strongest barrier	Less emphasis	Partial D: S showed complexity across ages/contexts; less prominent in I
Benefits & Objectives	Affirmed TR benefits	Affirmed TR benefits	C: Both confirmed TR advantages

### Ethical considerations

The study was approved by the Ethics Committee of the German Association for Physiotherapy at the Physio Academy (EK2022-02) and performed in accordance with the Declaration of Helsinki [32].

### Reflexivity

The research team consisted of a PT and academic assistant (MS) and an experienced post-doctoral researcher (AKR), providing both clinical and research perspectives. Member checking was employed to verify accurate representation of participants’ views.

## Results

### Survey participation and demographics

A total of 155 PTs agreed to participate in the survey, but data from three participants were incomplete or implausible, thus 152 participants were included in the final analysis. This sample represents approximately 0.12% of outpatient PTs in Germany.

The majority of respondents were female (65.1%, 99/152), with a mean age of 42 years (range 23–67, SD ± 12.5). Participants had an average of 16.7 years of professional experience (range 0–46 years, SD ± 12.0). The majority (60.5%, *n* = 92) graduated from vocational school, while 39.5% (*n* = 60) of the participants have an academic degree. Regarding their roles, 44.1% (*n* = 67) were practice owners, and 40.8% (*n* = 62) worked as staff PTs. A significant majority (83.9%, *n* = 128) worked in teams, primarily managing people with musculoskeletal conditions (87.7%, *n* = 133), followed by neurological conditions (30.9%, *n* = 47) and sports (25.7%, *n* = 39). Table 2 provides a summary of participants’ demographics.

**Table 2:** Demographics & characteristics

Characteristic	Survey (*n* = 152)	Interview (*n* = 9)
**Gender**		
Male	53 (34.9)	4 (44.4)
Female	99 (65.1)	5 (55.6)
Other	0 (0.0)	0 (0.0)
**Age in years**		
Mean (SD)	41 (± 12.5)	37 (± 11.9)
Range	22–66	23–59
**Work experience in years**		
Mean (SD)	17 (± 12.0)	13 (± 12.3)
Range	0–46	1–36
**Professional Qualification**		
Vocational Diploma	92 (60.5)	6 (66.7)
Academic Degree	60 (39.5)	2 (22.2)
• Bachelor	39 (25.7)	1 (11.1)
• Master	18 (11.8)	1 (11.1)
• PhD	3 (2.0)	0 (0.0)
**Professional Role**		
Staff Physiotherapist	60 (39.5)	6 (66.7)
Management Position	25 (16.4)	1 (11.1)
Practice Owner	67 (44.1)	2 (22.2)
**Clinical Specialty**†		
Musculoskeletal	133 (87.5)	9 (100.0)
Neurology	47 (30.9)	2 (22.2)
Cardiopulmonary	20 (13.2)	0 (0.0)
Pediatrics	16 (10.5)	2 (22.2)
Sports	39 (25.7)	1 (11.1)
Other	15 (9.9)	0 (0.0)
**Practice Size**		
Solo Practice	24 (15.8)	2 (22.2)
Team Practice	128 (84.2)	7 (77.8)
• 2–5 therapists	52 (40.6)	2 (28.6)
• 6–10 therapists	41 (32.0)	5 (71.4)
• >10 therapists	32 (25.0)	0 (0.0)

Note: Values are n (%) unless otherwise specified, † Multiple responses possible

### Use of TR (lockdown, current, future)

**Fig. 2:**
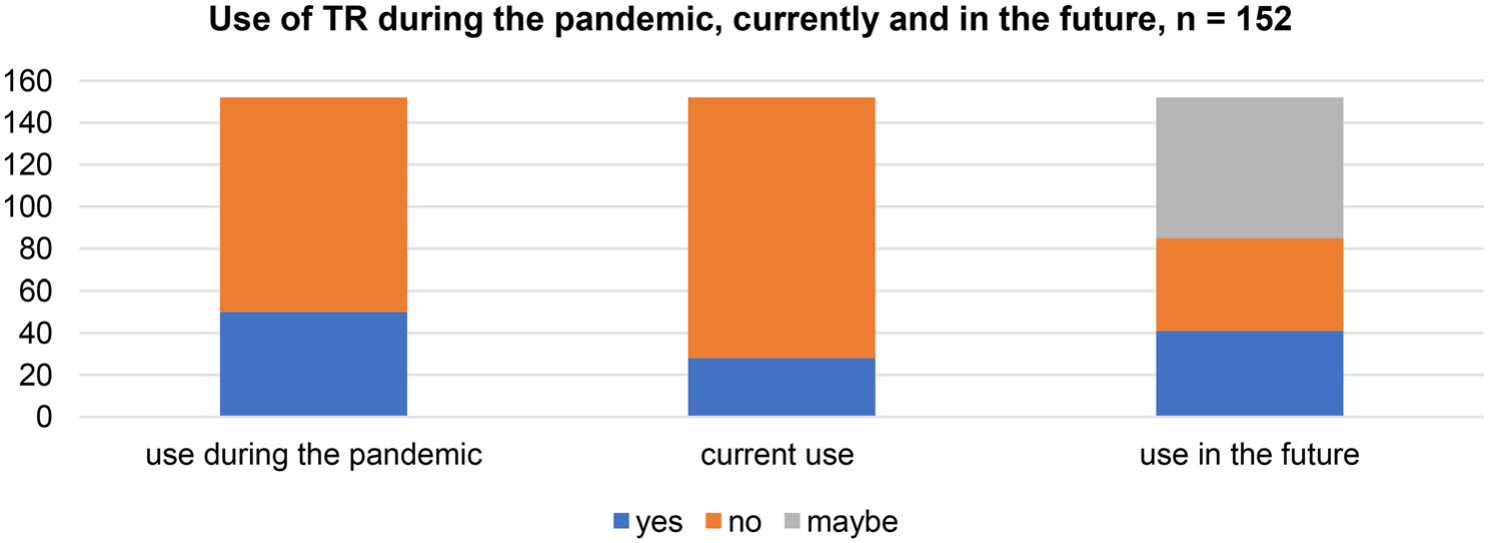
Use of video therapy (TR) during lockdown, currently and in the future; *n* = 152

The use of TR varied across different time periods. 32.26% (*n* = 50) of respondents reported that they used TR in the first COVID-19 lockdown. At the time of the survey, use had decreased to 18.06% (*n* = 28). 26.45% (*n* = 41) intended to use TR in the future, while 43.87% (*n* = 67) considered using it. Notably, 29.68% (*n* = 44) of respondents did not plan to use TR in the future. (Fig. 2)

### TR practices

Among TR users, the most common conditions treated were musculoskeletal (75%, 41/55), sports (38%, 21/55), pulmonology (33%, 18/55), and neurology (27%, 15/55). The majority of participants (64%, 34/53) used TR as an adjunct to face-to-face therapy, while 42% (23/53) used it as a standalone intervention.

**Fig. 3:**
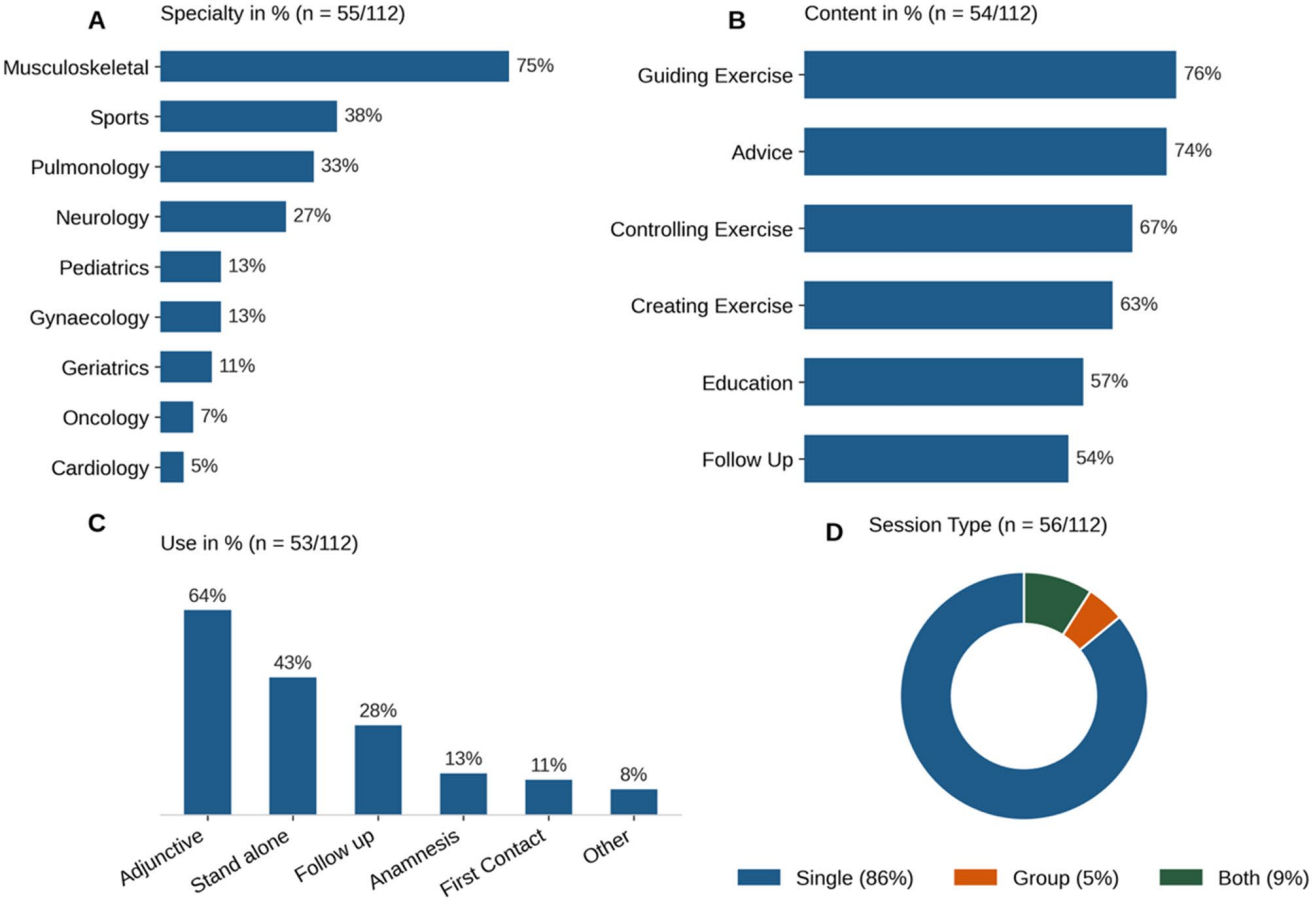
**A**) Percent of participants using TR for selected conditions; 55/112, no answer 57; **B**) Percentage on the extent of TR use; 53/112, no answer 59; **C**) Percentage of Content delivered in TR; 54/112, no answer 58; **D**) Percentage of settings in which TR is offered; 56/112, no answer 56

The primary content delivered through TR included guiding exercises (76%, 41/54) and providing advice (74%, 40/54). TR sessions were predominantly conducted individually (83%, 48/56), with only a small number of PTs offering group sessions (5%, 3/56) or both (9%, 5/56). Figure 3 illustrates these TR practices in a multi-panel layout. (Figure 3. TR Practices: speciality, content, use, and session types)

### Barriers to TR use

The adoption of TR among PTs faces several barriers, which can be understood through the lens of the TDF. In the domain of Role and Identity, the most significant barrier identified was the lack of physical examination, with 74% of respondents agreeing this was a major barrier.

Beliefs about Consequences and Capabilities emerged as the second most significant barrier, particularly concerning patients. Respondents expressed concerns about patients’ interest and technical capabilities for TR.

Within the Environmental Context and Resources domain, technical challenges and data protection concerns were generally not perceived as significant barriers. 70% of respondents disagreed that technical challenges were a major issue, while 55% disagreed that data protection was a significant concern. Survey data indicated that PTs perceived technical challenges on the patient side as a potential barrier to TR adoption. In terms of knowledge, personal knowledge gaps were not seen as a major barrier, with 59% of respondents disagreeing that this was a significant issue. However, 50% of respondents consider TR as less effective than treatment in practice.

**Fig. 4:**
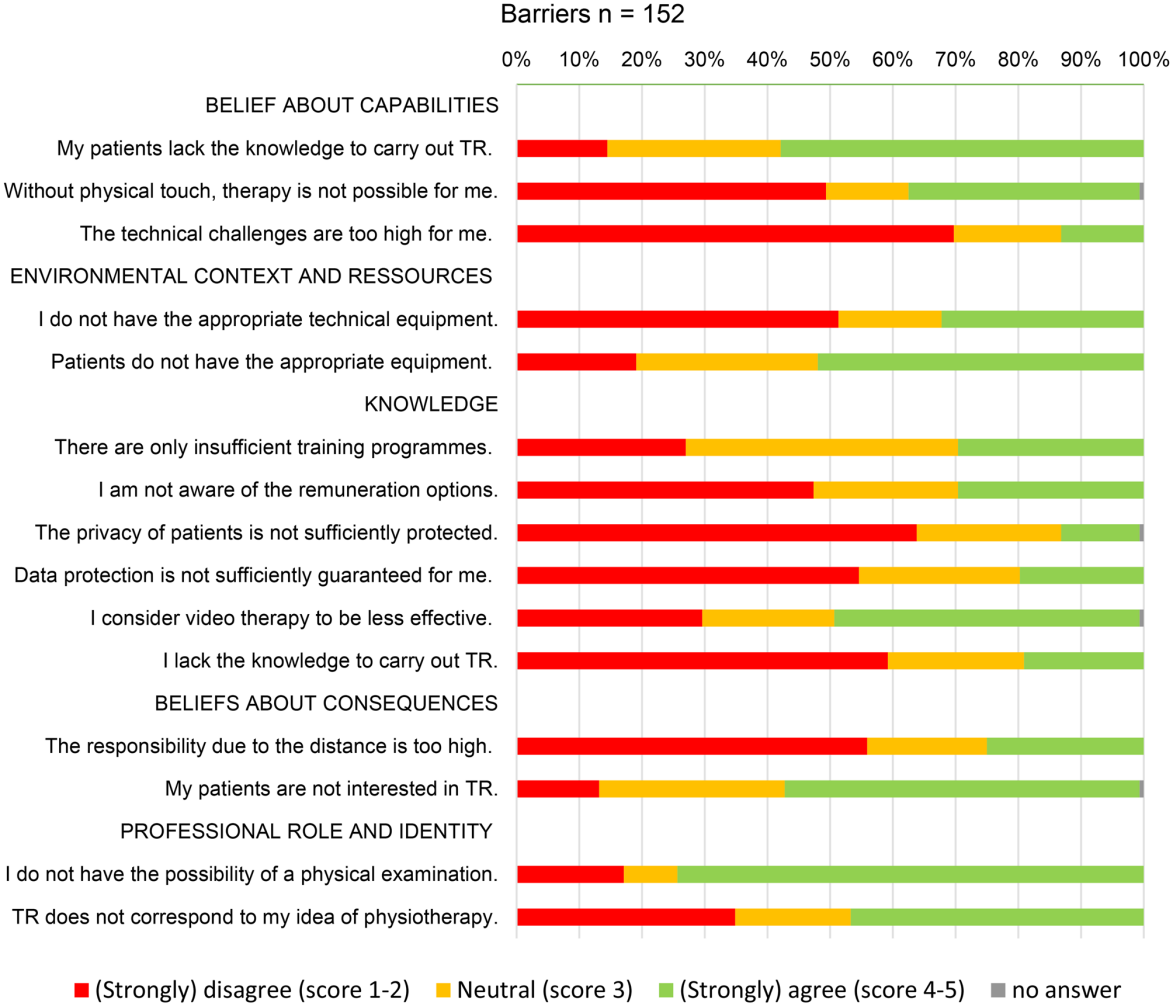
Survey data, reasons that hinder the use of TR, capitalized headlines indicate TDF (theoretical domains framework) categories

Figure 4 presents a summary of the main barriers to TR use, showing the percentage of agreement and disagreement for each potential barrier (Fig. 4).

### Facilitators for using TR

**Fig. 5:**
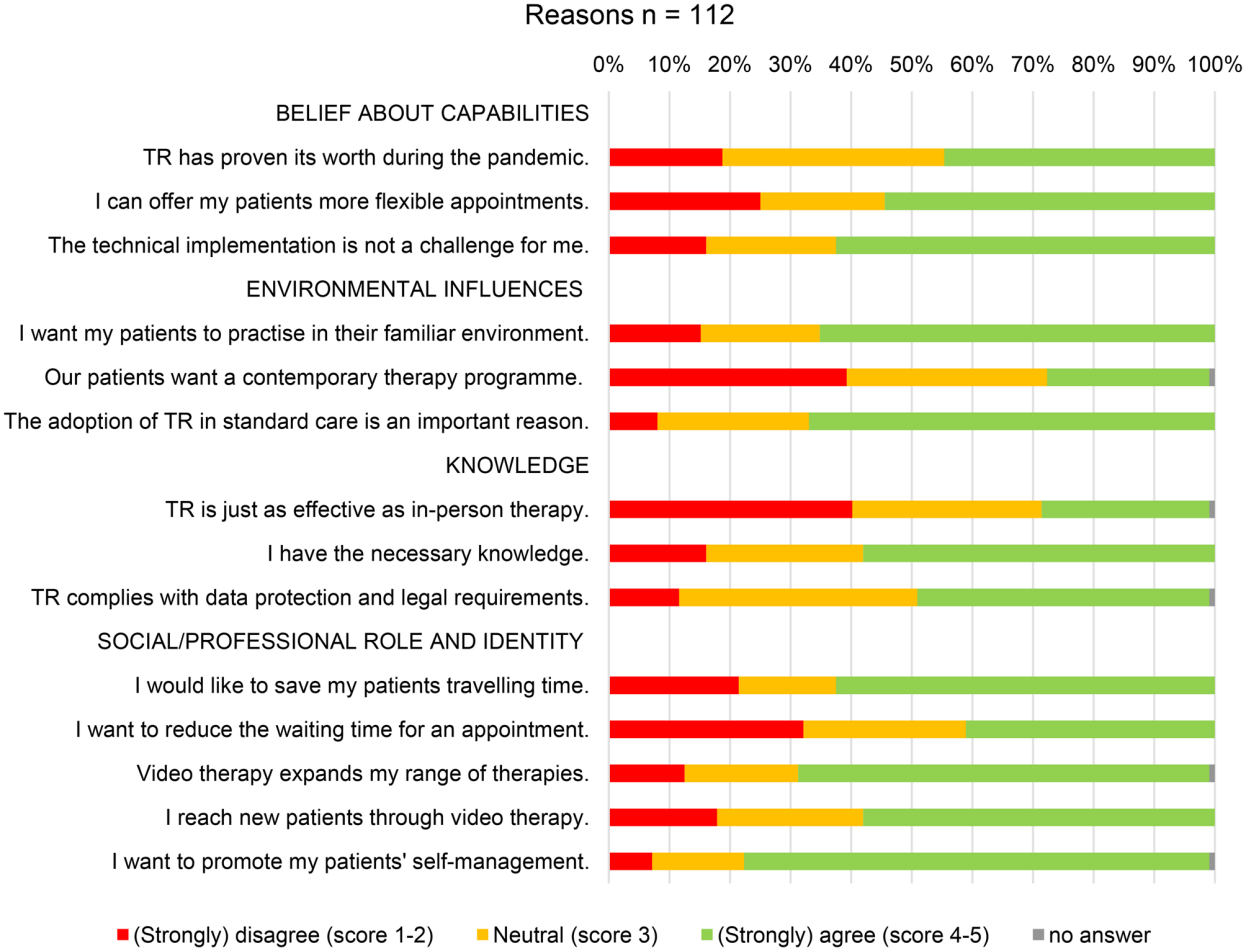
Survey data, reasons that facilitate the use of TR, capitalized headlines indicate TDF (Theoretical Domains Framework) categories

Among the participants who offered TR or were considering it (*n* = 112), several reasons for using TR were identified across multiple domains. Within the Role and Identity domain, promoting patient self-management was a key factor, with 78% of respondents in agreement. Additionally, 69% agreed that broadening the range of therapies by integrating remote options was important. Environmental influences also played a significant role, with the integration of the home setting into treatment (65% agreement) and the ability to bill via standard tariff (67% agreement) seen as particularly beneficial. However, some factors received more neutral responses from participants. These included the reduction of waiting time for appointments (falling under the Social Influences domain), the perceived efficacy of TR (Knowledge domain), and patients’ preference for contemporary services (Environmental Context and Resources domain). Figure 5 presents a summary of the main reasons to TR use, showing the percentage of agreement and disagreement for each potential facilitator.

### Interview results

Interviews were conducted with nine PTs (five female, four male) from four different German federal states. The mean age was 37.0 years (SD 11.9, range 23–59), and the mean work experience was 13.0 years (SD 12.3, range 1–36). Five participants were trained as PTs at a vocational school, while four received their training elsewhere. The interviews identified several barriers to TR implementation and provided insights into facilitating factors. Key themes that emerged from the analysis include:

### Barriers

#### PTs attitude

The qualitative data indicated that PTs attitudes and perceptions emerged as a notable factor influencing the implementation of TR, whereas patient-related barriers were less frequently cited by interview participants:

This has caused incredible problems for many of my colleagues because they have always worked ‘hands-on’ and are doing the same now (in relation to the time after the lockdown). (PT3)

#### Patient acceptance

Some patients who had encountered TR in practice were surprised by the positive results:

So a lot of people rated the experience with TR as very positive, they said they wouldn’t have thought that it would cover such a wide spectrum. (PT4)

#### Technical challenges

Insufficient internet bandwidth was emphasized as a significant technical challenge, not only in rural areas but also in cities:

My patients told me you’re frozen, you’re black, I can’t see you anymore, it’s pixelated. It was really nasty, especially in rural areas. It was really exhausting (PT1)

I also noticed the experience with the limitation in a city like Nuremberg (…) So that means certain times when I had therapy, you could forget that, nothing worked there (PT2)

#### Legal and liability concerns

The interviews revealed ambiguities regarding liability and legal frameworks:

The question is, if the 85-year-old actually falls, who is liable? (PT2)

#### Remuneration and financial considerations

Participants generally supported TR coverage by health insurance but criticized inflexible regulations. One interviewee doubted that the current remuneration would motivate larger physiotherapy chains to change their care model:

It is not worthwhile for this physiotherapy chain to invest in TR because they simply have enough patients anyway (PT5)

#### Organizational factors

Practical constraints, such as room allocation, were identified as potential barriers for practices:

So for us, we also allocate the rooms in the organisation, so to speak, and on Monday, because I’m still in the practice on Monday, it’s really very tight with the rooms, so it’s difficult (PT8)

#### Implementation approach

Interviewees emphasized the need for a professional and managed approach to TR implementation:

This also requires a lot of work from us and a clear and professional realization. (PT4)

#### Unexpected reservations

Notably, reservations about TR were not limited to older or less tech-savvy populations.:

Which personally also surprised me a lot, I actually mainly have young people with scoliosis and actually the reservations about the camera and the internet were relatively high (PT2)

#### Facilitators

PTs were asked about factors they believed would positively influence TR implementation. The results identified five categories of facilitators:

1. PT-related factors: Willingness and openness to new ways of working and the integration of digital technologies.
2. Infrastructure: Management or practice owners should provide ready-to-use technology and appropriate infrastructure, including scheduled remote therapies and dedicated spaces. A stable internet connection was deemed crucial for reducing problems during remote work.
3. Implementation process: PTs hypothesized that implementation would benefit from a clear, structured approach rather than ad hoc adoption. This includes careful planning and preparation, staff training and support, and gradual integration with existing workflows.
4. Flexibility: PTs expressed a preference for more autonomy in TR use, contrasting with current regulations that limit TR services to 50% of therapy sessions.
5. Patient involvement: PTs identified patient participation in the decision-making process as a strong facilitator for TR and other remote services.

### Integrating quantitative and qualitative findings

In this phase, we integrate the data gathered from the survey with the insights from the interviews, highlighting areas of convergence and divergence between the two data sources.

Key Insights:

1. Physical examination remains a complex issue, with differing perspectives from survey and interview data.
2. PTs attitudes emerged as a strong barrier in interviews, not captured by the survey.
3. Technical challenges, particularly internet bandwidth, are more significant than the survey indicated.
4. Legal and data protection concerns require further attention.
5. Remuneration’s impact on TR adoption is complex and requires more investigation.
6. Patient-related factors may be less of a barrier than initially thought based on survey data alone.
7. Both data sources agree on the potential benefits of TR implementation.

This integration reveals both convergences and divergences between quantitative and qualitative data, providing a comprehensive picture of barriers and facilitators in TR implementation. The divergences underscore the complexity of TR adoption in physiotherapy.

## Discussion

### Key findings

This study provides valuable insights into the use of TR among PTs in Germany. Our findings reveal that while TR use peaked during the COVID-19 lockdown, with 32.26% of PTs adopting this approach, its use has since decreased to 18.06%. It’s important to note that before the pandemic, TR treatments were not included in existing regulations and were therefore practically non-existent. The COVID-19 crisis led to a rapid adaptation of regulations, allowing therapy to be conducted wholly or partially via video. Although TR is now officially recognized and reimbursable, its use has decreased from peak levels. This suggests that while TR remains an established treatment option in Germany, both therapists and patients often prefer in-person treatment when available.

The primary barrier to TR adoption was the lack of physical examination, a concern shared by 74% of respondents participating in the survey. Interestingly, patient-related factors emerged as the second most significant barrier, with PTs expressing concerns about patients’ knowledge, interest, and technical capabilities regarding TR.

Despite survey results suggesting that technical challenges were not major barriers, our qualitative data painted a different picture, with interviewees reporting significant issues related to internet connectivity, even in urban areas. This discrepancy highlights the complex nature of TR implementation. On the positive side, the main motivations for using TR included its potential to promote patient self-management (78% agreement) and to broaden the range of available therapies (69% agreement).

Our study also uncovered some unexpected findings. Notably, younger patients showed surprising reservations about TR, particularly in treatments requiring undressing, challenging the assumption that younger individuals are universally more accepting of digital health solutions. Furthermore, organizational factors, such as scheduling and room allocation, emerged as significant challenges in the practical implementation of TR in physiotherapy practices.

### Comparison with existing literature

#### Implementation and use

The decline in TR use following the COVID-19 pandemic aligns with findings from other countries. Rettinger et al. (2021) observed a similar trend in Austria, although they noted a changing attitude towards teletherapeutic interventions [33]. Our finding of 18.06% current TR use is lower than rates reported in some other countries, such as Australia, where Bennell et al. (2021) found that 81% of PTs intended to continue using TR post-pandemic [16]. This disparity might be attributed to differences in healthcare systems, reimbursement policies, or cultural attitudes towards telehealth.

German PTs caution toward TR matches findings from Switzerland, where Rausch et al. (2021) found that 67.1% of PTs were skeptical about remote therapy as an alternative to in-person treatment [26]. Similar results were found in Austria [34]. This pattern suggests that TR implementation challenges among PTs may be common across German-speaking countries, possibly due to shared cultural or educational backgrounds that traditionally emphasize hands-on treatment approaches.

#### Barriers and facilitators

The primary barrier identified in our study - the lack of physical examination - was also identified from Malliaras et al. (2021), who reported similar concerns among allied health clinicians, emphasizing the perceived importance of hands-on therapy [19]. However, our study adds nuance to this understanding by highlighting that patient-related factors are the second most significant barrier from the PTs perspective, a finding not prominently featured in some previous studies. This suggests that successful implementation of TR may require not only addressing therapists’ concerns but also educating and engaging patients.

The unexpected finding of younger patients’ hesitancy towards TR, particularly in treatments requiring undressing, contrasts with the general assumption that younger individuals are more accepting of digital health solutions. This aligns with Barton et al.‘s (2022) mixed-methods evaluation, which found that acceptance of telehealth was not necessarily higher among younger patients with musculoskeletal pain [35]. Our findings suggest that privacy concerns and the nature of the treatment may play a more significant role in TR acceptance than age alone, highlighting the need for context-specific TR implementation to address individual factors.

Survey responses and interview findings differed regarding technical challenges, showing the complex nature of TR implementation. This highlights why mixed-methods research is valuable for understanding TR implementation challenges, as discussed by Caffery et al. (2017) [36]. It also suggests that the technical infrastructure for TR may be more problematic than initially perceived, potentially requiring targeted investments and support.

#### Benefits and applications

Our findings show that PTs view patient self-management as a key benefit of TR, aligning with current trends in physiotherapy. Hutting et al. (2019) highlighted how PTs can promote self-management, especially for patients with ongoing musculoskeletal conditions [37]. PTs in the interviews clearly support patient involvement through shared decision-making in whether TR could be a viable treatment option. This approach aligns with existing research on shared decision-making in healthcare. When applied in practice, shared decision-making improves patient-clinician communication and enhances patients’ understanding of the potential benefits and risks of interventions [38]. Moreover, it increases patient involvement in the decision-making process and their sense of being well-informed [38]. In the context of TR, this collaborative approach allows for a more personalized consideration of whether remote therapy is suitable for a patients’ specific needs, preferences, and circumstances. This aligns with the findings of Hanlon et al. (2017), who highlighted the potential of telehealth interventions in supporting self-management especially of long-term conditions [39]. Combining TR with shared decision-making and self-management techniques offers promising ways to improve care, especially for patients with chronic conditions.

Our finding that TR is predominantly used as an adjunct to face-to-face therapy (64% of users) rather than as a standalone intervention aligns with the hybrid model proposed by Baroni et al. (2023) for musculoskeletal physiotherapy [40]. This blended approach may offer a compromise that allows PTs to maintain hands-on elements of care while leveraging the benefits of TR, such as increased patient engagement and self-management.

The potential of TR to broaden the range of therapies and integrate the home environment into treatment, as identified in our study, aligns with the growing recognition of the importance of context in physiotherapy interventions. Wijma et al. (2017) emphasized the role of patient-centeredness in physiotherapy, and TR may offer unique opportunities to tailor treatments to patients’ home environments and daily routines [41].

#### Implications for practice and policy

The challenges in transitioning from hands-on to hands-off approaches reflect a broader shift in physiotherapy practice. TR could potentially catalyze this shift, encouraging the development of skills in exercise prescription, patient education, and coaching. This has implications for physiotherapy education and continuing professional development, suggesting a need to incorporate TR-specific skills into curricula and training programs.

Organizational factors, such as scheduling and room allocation, emerged as significant challenges, aligning with Wade et al.‘s (2014) findings on the importance of organizational readiness in sustaining telehealth services [42]. These findings emphasize the need for comprehensive implementation strategies that address practical challenges in the specific context of German physiotherapy practices. This need was also mentioned by PTs in the interviews.

The mixed perceptions regarding TR efficacy contrast with recent research showing promising results ([3, 9]. Research, particularly involving chronic patients, shows that one in two patients has a positive attitude towards telerehabilitation, and for over half of the patients, the experience was more positive than initially expected [43]. The effects on patient satisfaction are comparable between in-person and remote treatments across a variety of diagnoses, while TR even outperforms traditional methods in terms of attendance to appointments and exercise adherence [44].

However, a recent systematic review by Kaczorowski et al. concludes that there is still a lack of high-quality randomized controlled trials (RCTs) demonstrating the non-inferiority of teletherapeutic applications [45]. In particular, questions remain regarding delivery mode and effectiveness [45].

Despite these challenges, TR offers significant opportunities and should be considered for treatments. Patient groups with rare diseases, who often fly under the radar and have limited access to specialized care, could especially benefit [46]. For these patients, TR represents an option to increase accessibility to therapy.

The gap between therapists’ perceptions and research findings indicates a need for standardized guidelines in German physiotherapy. Such guidelines could reduce skepticism and help maintain consistent quality in TR services. Supporting this need, the American Physical Therapy Association (APTA) recently published clinical practice guidelines for TR in physical therapist practice [42]. These guidelines provide evidence-based recommendations for implementing TR across various patient populations and practice settings, potentially offering a valuable framework for developing similar standards in the German context.

While further research is needed, TR shows promise in improving patient care and should be explored as a complementary approach to traditional physiotherapy methods. Its potential to enhance accessibility and adherence makes it a valuable tool in the evolving landscape of healthcare delivery.

The slower adoption of TR in Germany compared to some other countries may reflect cultural factors, such as a traditional emphasis on hands-on care or privacy concerns. Understanding and addressing these cultural nuances will be crucial for the successful integration of TR into German physiotherapy practice.

### Practical implications

The findings from this study suggest several key approaches for implementing TR in physiotherapy practice. A structured implementation process should be established before introducing TR services. This includes setting up dedicated spaces, scheduling remote therapy sessions, and providing staff training on both technical aspects and remote therapy delivery.

The technical infrastructure forms an essential foundation. A stable internet connection is crucial, and practices need to prepare backup plans for technical difficulties. A professional setting with appropriate lighting and background should be established for video consultations.

Support for professional adaptation is necessary as PTs transition from hands-on to remote therapy approaches. Clear guidelines should outline which conditions are suitable for TR, and training should focus on remote assessment and monitoring techniques.

Patient engagement represents the final key component. Practices should develop methods to identify suitable candidates for TR and clearly communicate how remote therapy works. Decisions about combining in-person and remote sessions should be made together with patients to ensure the best possible care.

### Strengths and limitations

A key strength of this study is its mixed-methods design, which provided a comprehensive view of TR use in German physiotherapy practice. The integration of quantitative and qualitative data offered nuanced insights into barriers and facilitators of TR implementation, capturing both broad trends and individual experiences. This approach allowed us to identify discrepancies between survey responses and interview findings, particularly regarding technical challenges, highlighting the complex realities of TR implementation.

However, the study also has some limitations. A significant limitation is the survey response rate. With 152 participants representing only approximately 0.12% of outpatient PTs in Germany, the sample fell considerably short of the targeted 500 participants. This limited response may itself reflect the current status of TR in German physiotherapy practice, as some physiotherapists may not have participated believing that prior TR experience was required.

The small number of focus group interviews (*n* = 9) limits the generalizability of the qualitative findings to the broader population of German PTs. Additionally, the cross-sectional nature of the survey prevents identification of longitudinal trends or changes in attitudes and practices over time. The self-reported nature of the data may introduce bias, as participants’ responses could be influenced by social desirability or recall issues.

Reflexivity considerations are important to acknowledge. The primary investigator (MS) has clinical experience as a PT and has previously provided TR services, bringing a generally positive perspective toward technological integration in physiotherapy. While this experience provided valuable insights, it may have influenced data interpretation despite efforts to maintain neutrality. The second researcher (AKR), while not having direct TR experience, contributed research expertise from similar studies in Switzerland, offering a complementary analytical perspective. Steps were taken to minimize potential bias. Survey questions were deliberately formulated using neutral, open-ended language. The coding process primarily followed a predetermined framework focusing on the main research questions, with any disagreements resolved through researcher discussion. During focus group interviews, the primary investigator introduced himself as a neutral researcher and moderator, consciously setting aside his clinical experience to avoid influencing participant responses. These reflexivity considerations should be considered when interpreting the study findings.

A final limitation is the study’s exclusive focus on PTs perspectives, despite patients’ views and experiences being crucial for successful TR implementation.

### Future research directions

Future research should explore the effectiveness of TR for specific conditions in the German context, particularly in areas where our findings diverge from international trends. Longitudinal studies could provide insights into changing attitudes and practices over time. Additionally, identifying specific target groups that could benefit most from TR services in the German healthcare system is an important area for future investigation. This could help in tailoring TR interventions and implementation strategies to meet the needs of different patient populations.

Economic analyses specific to the German healthcare system are needed to support informed decision-making about TR adoption.

Crucially, research into patient experiences and preferences regarding TR in the German context would complement our findings and provide a more holistic understanding of TR implementation. This patient-centered perspective is essential for developing TR services that meet both clinical needs and patient expectations.

## Conclusion

The implementation of TR in German physiotherapy has growth potential but currently faces several barriers that need to be overcome. Successful implementation requires addressing several key factors related to PTs including: Their beliefs about capabilities and consequences (e.g., perceptions that patients lack the necessary skills or interest in TR), knowledge (e.g., understanding of telehealth technologies and best practices), their professional role and identity (e.g., adapting to a new mode of care delivery that may feel less “hands-on”), environmental contexts, resources and influences (e.g., access to reliable internet and appropriate technology). By addressing these factors, the field can leverage TR’s potential to enhance patient care quality (e.g., improving self-management skills and exercise adherence), increase service access (e.g., providing consistent care for chronic patients), and adapt to evolving healthcare delivery models (e.g., integrating TR with in-person care for a hybrid approach).

## Electronic Supplementary Material

Below is the link to the electronic supplementary material.

Supplementary Material 1

Supplementary Material 2

## Data Availability

The datasets used and/or analysed during the current study are available from the corresponding author on reasonable request.

## Abbreviations

APTA: American Physical Therapy Association
TDF: Theoretical Domains Framework
TR: Telerehabilitation

## References

[1] Seivert S, Badowski ME. The Rise of Telemedicine: Lessons from a Global Pandemic. EMJ 9 Februar. 2021;5(1):64–9.

[2] Scott Kruse C, Karem P, Shifflett K, Vegi L, Ravi K, Brooks M. Evaluating barriers to adopting telemedicine worldwide: A systematic review. J Telemed Telecare 1 Januar. 2018;24(1):4–12. 10.1177/1357633X16674087 PMC5768250 29320966

[3] Cottrell MA, Russell TG. Telehealth for musculoskeletal physiotherapy. Musculoskelet Sci Pract 1 August. 2020;48:102193. 10.1016/j.msksp.2020.102193 PMC7261082 32560876

[4] Beschluss des Gemeinsamen Bundesausschusses über eine Änderung der Heilmittel-Richtlinie. Maßnahmen der Heilmitteltherapie als telemedizinische Leistung (Videotherapie) und weitere Änderungen [Internet]. Verfügbar unter: https://www.g-ba.de/downloads/39-261-5079/2021-10-21_HeilM-RL_Videotherapie_BAnz.pdf.

[5] GKV Spitzenverband. Vereinbarung über die Ergänzung des Vertrages nach § 125 Absatz 1 SGB V (Physiotherapie). Apr 4, 2022.

[6] Horsley S, Schock G, Grona SL, Montieth K, Mowat B, Stasiuk K. u. a. Use of real-time videoconferencing to deliver physical therapy services: A scoping review of published and emerging evidence. J Telemed Telecare 1 Dezember. 2020;26(10):581–9. 10.1177/1357633X19854647 31213166

[7] Orlando JF, Beard M, Kumar S. Systematic review of patient and caregivers’ satisfaction with telehealth videoconferencing as a mode of service delivery in managing patients’ health. PLoS ONE. 2019;14(8):e0221848. 31469865 10.1371/journal.pone.0221848 PMC6716655

[8] Gemeinsamer Bundesausschuss GA. Pressemitteilung Gemeinsamer Bundesausschuss gemäß § 91 SGB V [Internet]. Okt 21, 2021. Verfügbar unter: https://www.g-ba.de/downloads/34-215-992/37_2021-10-21_Heilmittel-RL_Telemedizin.pdf.

[9] Seron P, Oliveros MJ, Gutierrez-Arias R, Fuentes-Aspe R, Torres-Castro RC, Merino-Osorio C. u. a. Effectiveness of Telerehabilitation in Physical Therapy: A Rapid Overview. Phys Ther 1 Juni. 2021;101(6):pzab053. 10.1093/ptj/pzab053 PMC7928601 33561280

[10] Langeard A, Bigot L, Maffiuletti NA, Moussay S, Sesboüé B, Quarck G et al. Non-inferiority of a home-based videoconference physical training program in comparison with the same program administered face-to-face in healthy older adults: the MOTION randomised controlled trial. Age Ageing. 1. March 2022;51(3):afac059. 10.1093/ageing/afac059 35290431

[11] Cottrell MA, O’Leary SP, Raymer M, Hill AJ, Comans T, Russell TG. Does telerehabilitation result in inferior clinical outcomes compared with in-person care for the management of chronic musculoskeletal spinal conditions in the tertiary hospital setting? A non-randomised pilot clinical trial. J Telemed Telecare 1 September. 2021;27(7):444–52. 10.1177/1357633X19887265 31771410

[12] Nelson M, Bourke M, Crossley K, Russell T. Telerehabilitation is non-inferior to usual care following total hip replacement - a randomized controlled non-inferiority trial. Physiotherapy June. 2020;107:19–27. 10.1016/j.physio.2019.06.006 32026820

[13] Molina-Garcia P, Mora-Traverso M, Prieto-Moreno R, Díaz-Vásquez A, Antony B, Ariza-Vega P. Effectiveness and cost-effectiveness of telerehabilitation for musculoskeletal disorders: A systematic review and meta-analysis. Annals Phys Rehabilitation Med 1 Februar. 2024;67(1):101791. 10.1016/j.rehab.2023.101791 38128150

[14] Ceprnja D, Clark T, Young J, Lee R, Flynn K, Maka K. Evaluating experiences, usability and patient satisfaction with telehealth for tertiary outpatient physiotherapy services during COVID-19: A mixed-methods study. Physiotherapy Theory Pract 6 April. 2022;0(0):1–9. 10.1080/09593985.2022.2059423 35387568

[15] Haines KJ, Sawyer A, McKinnon C, Donovan A, Michael C, Cimoli C. u. a. Barriers and enablers to telehealth use by physiotherapists during the COVID-19 pandemic. Physiotherapy March. 2023;118:12–9. 10.1016/j.physio.2022.09.003 PMC9450484 36308980

[16] Bennell KL, Lawford BJ, Metcalf B, Mackenzie D, van den Russell T. u. a. Physiotherapists and patients report positive experiences overall with telehealth during the COVID-19 pandemic: a mixed-methods study. J Physiother Juli. 2021;67(3):201–9. 10.1016/j.jphys.2021.06.009 PMC8188301 34147399

[17] Tyagi S, Lim DSY, Ho WHH, Koh YQ, Cai V, Koh GCH. u. a. Acceptance of Tele-Rehabilitation by Stroke Patients: Perceived Barriers and Facilitators. Archives Phys Med Rehabilitation Dezember. 2018;99(12):2472–e24772. 10.1016/j.apmr.2018.04.033 29902469

[18] Lawford BJ, Bennell KL, Kasza J, Hinman RS. Physical Therapists’ Perceptions of Telephone- and Internet Video-Mediated Service Models for Exercise Management of People With Osteoarthritis. Arthritis Care Res (Hoboken) März. 2018;70(3):398–408. 10.1002/acr.23260 28437566

[19] Malliaras P, Merolli M, Williams CM, Caneiro JP, Haines T, Barton C. „It’s not hands-on therapy, so it’s very limited: Telehealth use and views among allied health clinicians during the coronavirus pandemic. Musculoskelet Sci Pract April. 2021;52:102340. 10.1016/j.msksp.2021.102340 PMC7862900 33571900

[20] Reynolds A, Awan N, Gallagher P. Physiotherapists’ perspective of telehealth during the Covid-19 pandemic. Int J Med Inf Dezember. 2021;156:104613. 10.1016/j.ijmedinf.2021.104613 PMC8503965 34688969

[21] Richter B, Wattenberg I, Vollmer AL, Hornberg C, Wrede B, Lätzsch R. Die COVID-19-Pandemie als Chance für Teletherapie? – Eine Umfrage bei Vertreter*innen von Gesundheitsfachberufen. Gesundheitswesen. 3. August 2021;a-1537-8933. 10.1055/a-1537-8933 PMC11248726 34344047

[22] Niknejad N, Ismail W, Bahari M, Nazari B. Understanding Telerehabilitation Technology to Evaluate Stakeholders’ Adoption of Telerehabilitation Services: A Systematic Literature Review and Directions for Further Research. Archives Phys Med Rehabilitation 1 Juli. 2021;102(7):1390–403. 10.1016/j.apmr.2020.12.014 33484693

[23] Ivankova NV, Creswell JW, Stick SL. Using Mixed-Methods Sequential Explanatory Design: From Theory to Practice. Field Methods Februar. 2006;18(1):3–20.

[24] Tovin MM, Wormley ME. Systematic Development of Standards for Mixed Methods Reporting in Rehabilitation Health Sciences Research. Phys Ther 4 November. 2023;103(11):pzad084. 10.1093/ptj/pzad084 37672215

[25] Valerio MA, Rodriguez N, Winkler P, Lopez J, Dennison M, Liang Y. u. a. Comparing two sampling methods to engage hard-to-reach communities in research priority setting. BMC Med Res Methodol 28 Oktober. 2016;16(1):146. 10.1186/s12874-016-0242-z PMC5084459 27793191

[26] Rausch AK, Baur H, Reicherzer L, Wirz M, Keller F, Opsommer E. u. a. Physiotherapists’ use and perceptions of digital remote physiotherapy during COVID-19 lockdown in Switzerland: an online cross-sectional survey. Arch Physiother Dezember. 2021;11(1):18. 10.1186/s40945-021-00112-3 PMC8261812 34233763

[27] Harris PA, Taylor R, Thielke R, Payne J, Gonzalez N, Conde JG. Research electronic data capture (REDCap)--a metadata-driven methodology and workflow process for providing translational research informatics support. J Biomed Inf April. 2009;42(2):377–81. 10.1016/j.jbi.2008.08.010 PMC2700030 18929686

[28] Cane J, O’Connor D, Michie S. Validation of the theoretical domains framework for use in behaviour change and implementation research. Implement Sci 24 April. 2012;7:37. 10.1186/1748-5908-7-37 PMC3483008 22530986

[29] Kuckartz U, Rädiker S. Qualitative Inhaltsanalyse: Methoden, Praxis, Computerunterstützung: Grundlagentexte Methoden. 5. Auflage. Weinheim Basel: Beltz Juventa; 2022. 274 S. (Grundlagentexte Methoden).

[30] O’Cathain A, Murphy E, Nicholl J. Three techniques for integrating data in mixed methods studies. BMJ 17 September. 2010;341:c4587. 10.1136/bmj.c4587 20851841

[31] Fetters MD, Curry LA, Creswell JW. Achieving integration in mixed-methods designs-principles and practices. Health Serv Res Dezember. 2013;48(6 Pt 2):2134–56. 10.1111/1475-6773.12117 PMC4097839 24279835

[32] World Medical Association. World Medical Association Declaration of Helsinki: Ethical Principles for Medical Research Involving Human Subjects. JAMA 27 November. 2013;310(20):2191–4. 10.1001/jama.2013.281053 24141714

[33] Rettinger L, Klupper C, Werner F, Putz P. Changing attitudes towards teletherapy in Austrian therapists during the COVID-19 pandemic. J Telemed Telecare 11 Januar. 2021;1357633X:20986038. 10.1177/1357633X20986038 PMC10195684 33430678

[34] Seebacher B, Bergmann E, Geimer C, Kahraman T, Reindl M, Diermayr G. März. Factors influencing the willingness to adopt telerehabilitation among rehabilitation professionals in Austria and Germany: a survey comparing data before and during COVID-19. Disability and Rehabilitation. 12. 2024;46(6):1149–57. 10.1080/09638288.2023.2193428 36970941

[35] Barton CJ, Ezzat AM, Merolli M, Williams CM, Haines T, Mehta N. u. a. It’s second best: A mixed-methods evaluation of the experiences and attitudes of people with musculoskeletal pain towards physiotherapist delivered telehealth during the COVID-19 pandemic. Musculoskelet Sci Pract April. 2022;58:102500. 10.1016/j.msksp.2021.102500 PMC8777472 35074694

[36] Caffery LJ, Martin-Khan M, Wade V. Mixed-methods for telehealth research. J Telemed Telecare 1 Oktober. 2017;23(9):764–9. 10.1177/1357633X16665684 27591744

[37] Hutting N, Johnston V, Staal JB, Heerkens YF. Promoting the Use of Self-management Strategies for People With Persistent Musculoskeletal Disorders: The Role of Physical Therapists. J Orthop Sports Phys Ther 1 April. 2019;49(4):212–5. 10.2519/jospt.2019.0605 30931733

[38] Hoffmann T, Bakhit M, Michaleff Z. Shared decision making and physical therapy: What, when, how, and why? Braz J Phys Ther. 2022;26(1):100382. 35063699 10.1016/j.bjpt.2021.100382 PMC8784295

[39] Hanlon P, Daines L, Campbell C, McKinstry B, Weller D, Pinnock H. Telehealth Interventions to Support Self-Management of Long-Term Conditions: A Systematic Metareview of Diabetes, Heart Failure, Asthma, Chronic Obstructive Pulmonary Disease, and Cancer. J Med Internet Res 17 Mai. 2017;19(5):e172. 10.2196/jmir.6688 PMC5451641 28526671

[40] Baroni MP, Jacob MFA, Rios WR, Fandim JV, Fernandes LG, Chaves PI. u. a. The state of the art in telerehabilitation for musculoskeletal conditions. Archives Physiotherapy 4 Januar. 2023;13(1):1. 10.1186/s40945-022-00155-0 PMC9810517 36597130

[41] Wijma AJ, Bletterman AN, Clark JR, Vervoort SCJM, Beetsma A, Keizer D. u. a. Patient-centeredness in physiotherapy: What does it entail? A systematic review of qualitative studies. Physiotherapy Theory Pract 2 November. 2017;33(11):825–40. 10.1080/09593985.2017.1357151 28820617

[42] Wade VA, Eliott JA, Hiller JE. Clinician Acceptance is the Key Factor for Sustainable Telehealth Services. Qual Health Res 1 Mai. 2014;24(5):682–94. 10.1177/1049732314528809 24685708

[43] Lawford BJ, Bennell KL, Kimp A, Campbell PK, Hinman RS. Understanding Negative and Positive Feelings About Telerehabilitation in People With Chronic Knee Pain: A Mixed-Methods Study. J Orthop Sports Phys Ther September. 2024;54(9):1–14. 10.2519/jospt.2024.12383 39207737

[44] Simmich J, Ross MH, Russell T. Real-time video telerehabilitation shows comparable satisfaction and similar or better attendance and adherence compared with in-person physiotherapy: a systematic review. J Physiotherapy Juli. 2024;70(3):181–92. 10.1016/j.jphys.2024.06.001 38879432

[45] Kaczorowski S, Donath L, Owen PJ, Saueressig T, Mundell NL, Topp M. Telemedicine for Patients with Musculoskeletal Pain Lacks High-Quality Evidence on Delivery Modes and Effectiveness: An Umbrella Review. Telemedicine and e-Health [Internet]. 21. Dezember 2023 [zitiert 24. Dezember 2023]; Verfügbar unter: https://www.liebertpub.com/doi/10.1089/tmj.2023.0255. 10.1089/tmj.2023.0255 38117672

[46] Lavorgna L, Maida E, Reinhard C, Cras P, Reetz K, Molnar MJ. u. a. The Growing Role of Telerehabilitation and Teleassessment in the Management of Movement Disorders in Rare Neurological Diseases: A Scoping Review. Telemed J E Health September. 2024;30(9):2419–30. 10.1089/tmj.2023.0702 38946606

