# Supplementary material for: Barriers and facilitators to telerehabilitation implementation: a mixed-methods study of German physiotherapists
Source: BMC Health Serv Res. 2026 May 9;26:671. doi: 10.1186/s12913-026-14636-6 (PMC13157660; doi:10.1186/s12913-026-14636-6)
Supplement: Supplementary file 1 — Supplementary Material 1 [file 12913_2026_14636_MOESM1_ESM.docx]

**Supplementary Material 2**

**Focus Group Agenda**

**Welcome by the Moderator**

- Consent for video and audio recording

**Introduction**

- For this study, I conducted a nationwide survey over three months in the summer on the use of video therapy in German physiotherapy. A total of 155 physiotherapists participated and shared their assessments. You participated in this survey and are familiar with its contents. Additionally, you have agreed to participate in today's group discussion, which I am very pleased about. Building on the nationwide survey, today's discussion aims to achieve the following:
  - Besides your personal assessment and opinion on video therapy in physiotherapy, I am particularly interested in the following aspects:
    1. Which person and patient groups do you consider suitable for video therapy?
    2. Which content would you most likely want to convey via video?
    3. In which phase would you use video-based communication?
  - These three questions focus on the benefits of video-based therapy and, more generally, on video communication with patients and clients.
- In the second block, I would like to discuss the obstacles to video therapy with you. What could prevent its use? What barriers do you see in the healthcare structure and within yourselves?
- The third block will focus on solutions. We want to discuss what could help you implement video-based communication with patients. We also want to talk about the benefits it could have for care if remote communication becomes an integral part of physiotherapy.

**Objective of the Work**

- My aim is to describe the current benefits of video therapy in Germany with the survey and two group discussions with physiotherapists and to critically place it in the context of worldwide usage. I want to collect, describe, and better understand the obstacles to its use. The goal of the discussion is to identify and document the factors that promote the use of video therapy. What conditions need to be met for the successful use of video communication?

**Icebreaker Question with a Brief Personal Introduction (each person responds)**

- Main questions discussed for 15 minutes per block
- Summary of what was said, especially the core questions: benefits, barriers, promoting factors

**Ground Rules**

- Role of the moderator: to lead the discussion but not participate in it. May interrupt to ensure everyone has a chance to speak or to steer the discussion in a new direction.
- All positions are important – agreement is not necessary; controversies are welcomed.
- Anyone can raise questions on the topic.
- I can present results from the survey upon request at any time.
- Responses should also be seen as further food for thought.
- The discussion will be recorded and subsequently anonymized – confidentiality is guaranteed – how much participants disclose is up to them.

**Guiding Questions and Detailed Questions**

**(1) Icebreaker + Introduction**

- Who are you, and what do you think about video therapy with patients?
- Do video consultations occur in your daily routine? When and with whom?
- How does it proceed?

**(2) For which patient groups could video therapy be beneficial?**

- MSK, Sports, Pulmonology, Neurology
- Are there specific patient groups that particularly come into question?
- Are there patient groups that do not come into question at all?
- How do you justify your selection?

**(3) What content is well-suited for video therapy?**

- Initiation of exercises
- Consultation
- Monitoring
- Creation of training plans
- Can the same content be conveyed via video as in person?
- Are there contents that are particularly well-suited for video?
- Does video have an advantage over contents conveyed in person?

**(4) In Germany, the use of video therapy is limited to 50% of treatment time according to the Heilmittelkatalog. The first and last appointments must take place in the practice. Are these regulations a hurdle or a sensible limitation?**

- As an accompaniment to therapy
- Comprehensive
- Follow-up care
- Which design of video therapy do you prefer?
- In which phases is video therapy sensible?
- Do all patients need the same?

**(5) Reasons for not considering video therapy are:**

- The patients
- The lack of physical examination, touch, and the general manner
- Knowledge of effectiveness
- Are these also your hurdles?
- How do you assess the mentioned hurdles?
- Do you agree with this?
- Is there anything that surprises you?
- Which reasons are highly significant, and which have less influence?

**(6) The survey shows that obstacles increase with age. Is it just a matter of time before video therapy really takes off?**

- Is video therapy a future topic?

**(7) It also seems to matter who makes the decision for/against video therapy and what support interested therapists receive. How should a well-made video therapy be structured in your opinion?**

- Who takes responsibility?
- Who performs it?
- What resources need to be created?

**(8) What reasons do you see for the use of video therapy?**

- Survey:
  - Reasons/Motivation
  - Promote self-management (1, 437)
  - Expand therapy spectrum (2, 410)
  - Billing via HL / standard care (3, 388)
  - Generate new patients (4, 386)
  - Save patients travel time (5, 373)
- What are your reasons from a physiotherapist's perspective?
- What would be reasons from a patient's perspective?

**(9) Conclusion (1 min Flashlight)**

- What is the most important thing we talked about today for you?
- What advice would you give to decision-makers dealing with the implementation of video therapy?
